# Global disability-adjusted life years and deaths attributable to child and maternal malnutrition from 1990 to 2019

**DOI:** 10.3389/fpubh.2024.1323263

**Published:** 2024-01-18

**Authors:** Rong Liu, Lucheng Pi, Fangqun Leng, Qing Shen

**Affiliations:** ^1^School of Public Health, Hangzhou Medical College, Hangzhou, China; ^2^Shenzhen Bao’an Chinese Medicine Hospital, Guangzhou University of Chinese Medicine, Shenzhen, China; ^3^West China School of Public Health and West China Fourth Hospital, Sichuan University, Chengdu, China

**Keywords:** trends, sub-Saharan Africa, low SDI, global burden of disease, child and maternal malnutrition

## Abstract

**Background:**

Child and maternal malnutrition (CMM) caused heavy disability-adjusted life years (DALY) and deaths globally. It is crucial to understand the global burden associated with CMM in order to prioritize prevention and control efforts. We performed a comprehensive analysis of the global DALY and deaths attributable to CMM from 1990 to 2019 in this study.

**Methods:**

The age-standardized CMM related burden including DALY and death from 1990 to 2019 were accessed from the Global Burden of Disease study 2019 (GBD 2019). The changing trend were described by average annual percentage change (AAPC). The relationship between sociodemographic factors and burden attributable to CMM were explored by generalized linear model (GLM).

**Results:**

Globally, in 2019, the age-standardized DALY and death rates of CMM were 4,425.24/100,000 (95% UI: 3,789.81/100,000–5,249.55/100,000) and 44.72/100,000 (95% UI: 37.83/100,000–53.47/100,000), respectively. The age-standardized DALY rate (AAPC = −2.92, 95% CI: −2.97% to −2.87%) and death rates (AAPC = −3.19, 95% CI: −3.27% to −3.12%) presented significantly declining trends during past 30 years. However, CMM still caused heavy burden in age group of <28 days, Sub-Saharan Africa and low SDI regions. And, low birth weight and short gestation has identified as the primary risk factors globally. The GLM indicated that the highly *per capita* gross domestic product, *per capita* current health expenditure, physicians per 1,000 people were contributed to reduce the burden attributable to CMM.

**Conclusion:**

Although global burden attributable to CMM has significantly declined, it still caused severe health burden annually. To strengthen interventions and address resources allocation in the vulnerable population and regions is necessary.

## Introduction

Despite significant progress have been achieved in improving global poverty and food security during past few decades, child and maternal malnutrition (CMM) still affects millions of women and children worldwide. It estimated that over 122 million people are experiencing hunger due to the pandemic, economic stagnation, climate change, and conflicts, with the Global Hunger Index (GHI) stands at a moderate 18.3 in 2023. Particularly, South Asia and Africa South of the Sahara are confronting severe hunger (GHI score 27.0). Africa South of the Sahara is experiencing the highest level of undernourishment at 21.7% (1). In the UNICEF-WHO low birthweight estimates for the 2023 edition, it is estimated that 19.8 million newborns were classified as low birthweight globally (2). Furthermore, 148.1 million children under the age of 5 were stunted, and 45 million were wasted (3). Previous study also highlights the significant impact on children in Sub-Saharan Africa, with a prevalence of 19.1% stunting in Senegal and 54.6% in Burundi of 2020 (4).

The World Health Assembly endorsed a comprehensive plan to reduce low birth weight, childhood stunting and wasting, anemia in women, and to increase exclusive breastfeeding during the first 6 months of life (5). However, there remains a significant gap while implementation to achieve the 2030 Sustainable Development Goal (SDG) targets. Given the current progress, it is projected that the 2030 target will be missed by 39.5 million children suffering from stunting (3). According to estimation, more than 20 million newborns are affected by low birth weight every year, and anemia rates among girls and women of childbearing age still remains worrisome. Moreover, anemia rates among women also remain high and are increasing in many countries (5). About 60% of children under the age of 5 years are anemic (with a higher proportion among children aged 6–24 months) in LMICs (6). Additionally, the prevalence of vitamin A deficiency and zinc deficiency remains stubbornly high in many regions and should not be ignored.

The evidence have shown that CMM increase the risk of negative health outcomes, causes heavy disability-adjusted life years (DALYs) and high mortality rates, particularly in low-and middle-income countries (LMICs) (7–10). It concluded that maternal malnutrition was associated with maternal anemia, mortality, brain defects, neuropsychiatric disorders, altered cognition, visual impairment, and motor deficits (2). Currently, more than half of all deaths of children under five can be attributed to malnutrition in globally (9, 10). For example, malnutrition accounts for 68.2% of total deaths in children under the age of 5 years, and also serves as the leading risk factor for health loss in all age groups, accounting for 17.3% of total DALY in India (11). In Africa, the prevalence of malnutrition among maternal was 23.5% (12), moreover, hunger is responsible for 45% of all childhood mortality (13). According to another national report from Papua New Guinea, roughly 33% of all hospital deaths among children under the age of 5 are directly or indirectly attributed to malnutrition (14).

Previous CMM studies have assessed its prevalence and trends (4, 15), its influence on health outcomes (16), associate factors (17), effective interventions (7), and the GBD-related study in specific region (11). Tracking the burden and changing trend attributed to CMM by sex, age, social development index (SDI), risk factors, and location can help countries/regions in comprehending the present status and advancements in addressing CMM-related diseases. This understanding can assist local governments, international organizations, civil society organizations and other stakeholders in identifying priorities for preventing and controlling of CMM. However, there is a lack of comprehensive studies from an integrated perspective. Therefore, this study aimed to evaluate the global burden and trends of disease attributable to CMM from 1990 to 2019.

## Materials and methods

### Data sources

#### The burden of disease attributable to child and maternal malnutrition

The data of burden attributable to CMM used in this study were derived from the GBD 2019,^1^ which is collected and analyzed by a consortium of more than 9,000 researchers. The data are collected from various sources, including census, household surveys, civil registration, disease registration, national health service records, air pollution surveillance, satellite imaging, cause of death testing and reporting, and high-quality literature (18, 19). GBD 2019 employs comprehensive methods to quantify health losses such as years of life lost (YLLs), years lived with disability (YLDs), DALY, and death of 87 risk factors in 204 countries and territories from 1990 to 2019. The following data were included in this study: DALY and death cases, age-standardized rates of DALY and age-standardized rates of death (ASDR) attributable to CMM by sex, 5 SDI quintiles (high, high-middle, middle, low-middle, and low SDI), and 21 GBD regions.

#### The covariates associated with child and maternal malnutrition

We aggregated a list of country-level covariates hypothesized to be directly or indirectly associated with the burden of CMM, including Gross Domestic Product (GDP) *per capita*, current health expenditure *per capita*, population, urban population (% of total population), population density, and physicians per 1,000 people (20).

#### The definition of specific risk factors of child and maternal malnutrition in GBD 2019

In GBD 2019, CMM includes six following specific risk factors: low birth weight and short gestation, child growth failure, suboptimal breastfeeding, iron deficiency, vitamin A deficiency and zinc deficiency. Low birth weight refers to any birth weight less than the population level birth weight TMREL (the birth weight that minimizes risk), and short gestation refers to all gestational ages below the gestational age TMREL. Stunting, wasting, and underweight were used to estimate child growth failure based on the growth standards for children aged 0–59 months by WHO (21). Suboptimal breastfeeding includes two distinct categories: non-exclusive breastfeeding (children under 6 months of age who are not exclusively breastfeed) and discontinued breastfeeding (children between 6 and 23 months) (22). Iron deficiency is defined as inadequate iron to meet the body’s needs (IDA women had a hemoglobin concentration lower than 115 g/L, 100 g/L for children) (23), while vitamin A deficiency is defined as serum retinol <70 μmol/L for women, 50 μmol/L for children. Zinc deficiency is defined as blood test below 0.60 mcg/mL (24).

### Statistics analysis

#### Join-point regression analysis

The join-point regression model, developed by U.S Surveillance Research Program of the National Cancer Institute (25), is utilized to identify the join-point in time series data. It conducts statistical modeling and analysis to determine the timing of trend changes and the changes in slope at these points, revealing critical moments of data change and patterns of trend variation (26).

In this study, join-point regression model was used to examine the changing trend of the age-standardized DALY rate and ASDR by sex, age, SDI, specific risk factor and geographical region from 1990 to 2019. The regression model was as follow: *ln* (age-standardized rate of DALY or death) = *α* + *β*X + *ε*, where X for the calendar year (27). The average annual percentage change (AAPC) was calculated by 100 × [exp (*β*) − 1]. When AAPC tested significantly greater than zero, the trend was defined as increased, by the contrary, it was defined the decreased trend. Meanwhile, we used “stable” to describe non-significant AAPC trend.

#### Generalized linear model

Generalized Linear Model is an extension of linear models, consisting of dependent variables, linear components, and link functions, and is applied to various types of data and scenarios. In this study, the log-linear generalized linear model was used to fit the age-standardized rates of DALY and ASIR and sociodemographic factors. The details were as follows: Log (ASR) = *β_0_* + *β_1_* * Log *per capita* GDP + *β_2_* * Log *per capita* current health expenditure + *β_3_* * Urban population (% of total population) + *β_4_* * Log Population + *β_5_* * Log Population density + *β_6_* * Log physicians (per 1,000 people). The best-fitting model was determined according to the Akaike information criterion (28).

## Results

### Global burden

**Figure 1:**
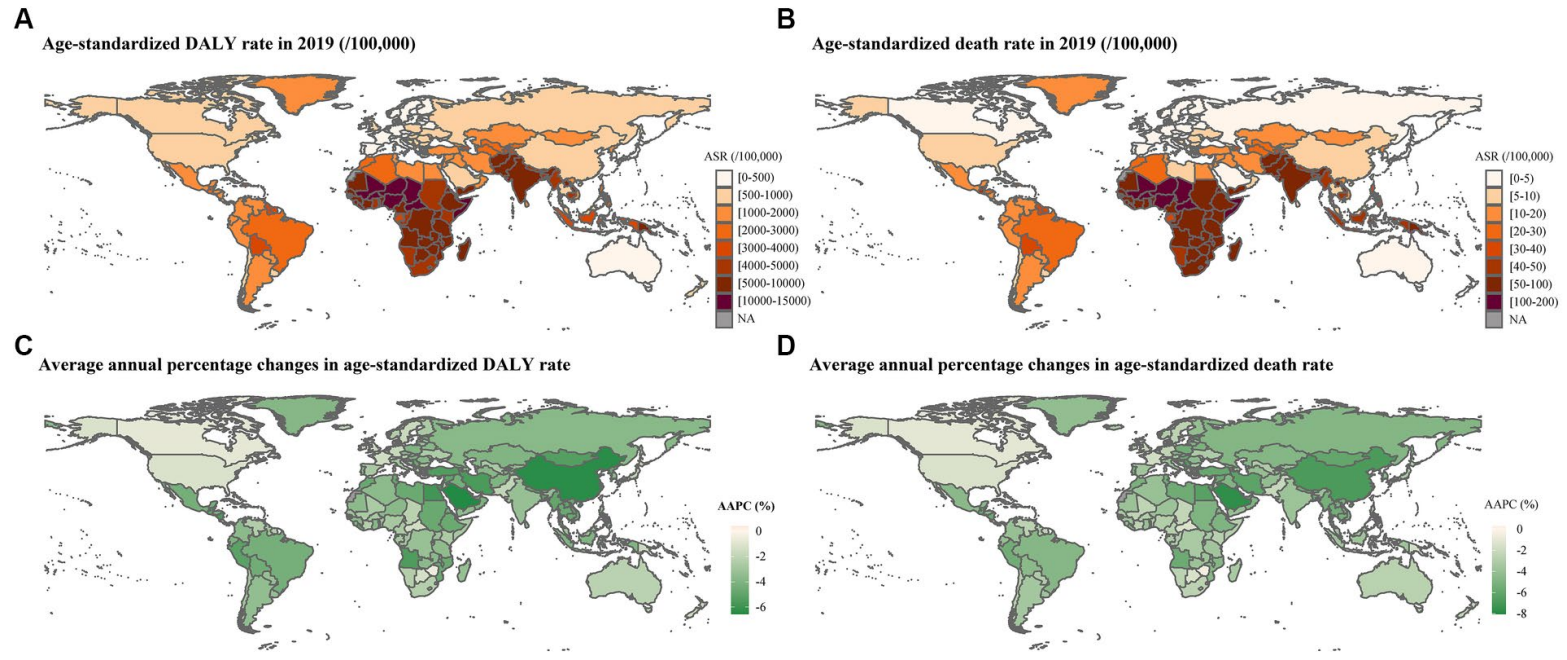
Global burden attributable to CMM in 2019 and average annual percentage change of age-standardized rates from 1990 to 2019. **(A)** Age-standardized DALY rate in 2019; **(B)** ASDR in 2019; **(C)** AAPC of age-standardized DALY rate from 1990 to 2019; **(D)** AAPC of ASDR from 1990 to 2019. ASR: age-standardized rate. An AAPC less than 0 indicates a significant decrease, an AAPC equal to 0 signifies stability, and an AAPC greater than 0 represents a significant increase.

Globally, there were 294,779,309 (95% UI: 252,994,742–349,534,865) DALY cases and 2,937,804 (95% UI: 2,489,636–3,512,073) death cases attributable to CMM in 2019. The global age-standardized DALY and death rates of CMM were 4,425.24/100,000 (95% UI: 3,789.81/100,000–5,249.55/100,000) and 44.72/100,000 (95% UI, 37.83/100,000–53.47/100,000), respectively (Table 1; Supplementary Table 1; Figures 1A,B).

**Table 1:** The age-standardized rates of DALY and death attributable to CMM in 1990 and 2019.

	Age-standardized rate of DALY (/100,000)		AAPC (%, 95 CI)	Age-standardized rate of death (/100,000)		AAPC (%, 95 CI)
Variables	1990	2019		1990	2019	
Global	10,394.11	4,425.24	−2.92	113.94	44.72	−3.19
	(9,572.24–11,343.22)	(3,789.81–5,249.55)	(−2.97 to −2.87)*	(104.75–124.75)	(37.83–53.47)	(−3.27 to −3.12)*
Gender						
Male	10,580.94	4,552.18	−2.88	117.06	46.96	−3.11
	(9,634.34–11,560.53)	(3,865.31–5,418.67)	(−2.93 to −2.83)*	(106.46–128.14)	(39.38–56.97)	(−3.19 to −3.04)*
Female	10,189.30	4,283.01	−2.95	110.85	42.33	−3.29
	(9,323.04–11,121.24)	(3,696.04–5,054.8)	(−3.03 to −2.87)*	(101.08–121.64)	(36.02–50.26)	(−3.37 to −3.21)*
Age group						
< 28 days	2,671,763.99	1,554,055.35	−1.88	30,041.66	17,464.36	−1.88
	(2,488,001.04–2,867,782.86)	(1,322,830.84–1,845,976.9)	(−1.99 to −1.77)*	(27,973.96–32,248.37)	(14,860.36–20,754.84)	(−1.99 to −1.77)*
28–364 days	158,943.50	43,824.56	−4.36	1,774.03	474.71	−4.46
	(141,990.37–177,390.18)	(35,699.95–54,831.29)	(−4.55 to −4.17)*	(1,585.77–1,984.12)	(382.27–598.54)	(−4.62 to −4.31)*
1–4 years	32,153.62	7,933.11	−4.71	356.5	78.05	−5.1
	(27,847.02–37,231.14)	(6,372.28–9,799.67)	(−4.86 to −4.57)*	(307.84–416.66)	(61–99.5)	(−5.22 to −4.99)*
5–14 years	1,095.85	928.37	−0.58	2.71	0.55	−5.44
	(826.46–1,436.3)	(675.19–1,264.03)	(−0.63 to −0.53)*	(1.92–3.94)	(0.44–0.67)	(−5.75 to −5.14)*
15–49 years	634.25	495.71	−0.81	3.66	1.41	−3.29
	(472.41–813.62)	(373.38–636.69)	(−1.03 to −0.59)*	(1.89–5.36)	(0.72–2.13)	(−3.95 to −2.63)*
50–69 years	664.92	459.77	−1.3	4.84	1.53	−3.79
	(498.11–868.03)	(342.5–605.37)	(−2.18 to −0.4)*	(3.76–6.5)	(1.36–1.74)	(−6.98 to −0.49)*
70+ years	880.1	564.34	−1.52	31.61	16.66	−2.13
	(723.61–1,063.16)	(453.77–709.82)	(−2.52 to −0.5)*	(26.68–36.51)	(14.6–18.13)	(−4.01 to −0.21)*
SDI region						
High SDI	1,090.72	580.86	−2.13	9.81	4.43	−2.69
	(1,005.07–1,191.91)	(517.96–649.96)	(−2.26 to −2)*	(9.21–10.54)	(4–4.89)	(−2.81 to −2.58)*
High–middle SDI	3,742.32	1,102.10	−4.12	38.19	9.13	−4.81
	(3,367.93–4,144.34)	(953.85–1,269.95)	(−4.24 to −4.00)*	(34.23–42.38)	(7.91–10.58)	(−4.96 to −4.65)*
Middle SDI	7,036.81	2,303.07	−3.79	77.47	22.58	−4.19
	(6,428.73–7,667.28)	(1,985.22–2,682.4)	(−3.93 to −3.65)*	(71.13–84.15)	(19.49–26.6)	(−4.34 to −4.04)*
Low–middle SDI	15,587.79	5,560.20	−3.49	174.34	55.06	−3.89
	(14,329.3–16,994.59)	(4,788.68–6,525.94)	(−3.61 to −3.37)*	(159.61–192.16)	(47.11–65.2)	(−4.31 to −3.45)*
Low SDI	21,174.88	8,411.63	−3.13	243.82	90.03	−3.37
	(19,010.44–23,558.42)	(7,020.74–10,230.56)	(−3.19 to −3.07)*	(218.06–271.71)	(74.34–110.95)	(−3.45 to −3.3)*
Type of Cause						
Suboptimal breastfeeding	729.36	201.77	−4.35	8.2	2.25	−4.38
	(538.09–913.91)	(143.44–270.38)	(−4.45 to −4.25)*	(6.06–10.29)	(1.59–3.03)	(−4.48 to −4.28)*
Iron deficiency	541.7	417.98	−0.89	1.33	0.53	−3.09
	(374.2–752.19)	(284.48–589.45)	(−0.91 to −0.87)*	(0.49–2.13)	(0.19–0.89)	(−3.3 to −2.87)*
Vitamin A deficiency	316.35	48.89	−6.25	3.25	0.36	−7.32
	(57.02–633.38)	(19.5–83.62)	(−6.47 to −6.03)*	(0.3–6.94)	(0.04–0.75)	(−7.59 to −7.06)*
Zinc deficiency	24.88	3.86	−6.2	0.28	0.04	−6.36
	(8.35–48.86)	(1–8.9)	(−6.4 to −5.99)*	(0.09–0.56)	(0.01–0.1)	(−6.57 to −6.15)*
Child growth failure	5,274.62	1,347.81	−4.59	62.5	15.93	−4.6
	(4,671.53–5,973.36)	(1,087.17–1,678.64)	(−4.77 to −4.41)*	(55.31–70.84)	(12.94–19.67)	(−4.89 to −4.32)*
Low birth weight and short gestation	4,376.50	2,604.41	−1.8	48.26	27.87	−1.9
	(4,073.15–4,688.56)	(2,232.22–3,085.68)	(−1.9 to −1.69)*	(44.9–51.81)	(23.73–33.09)	(−2.01 to −1.79)*
Southeast Asia, east Asia, and Oceania						
Southeast Asia	9,036.88	2,635.73	−4.16	104.1	29.05	−4.31
	(8,188.44–10,012.69)	(2,243.32–3,058.62)	(−4.24 to −4.09)*	(94.47–115.09)	(24.77–33.57)	(−4.42 to −4.2)*
East Asia	5,242.02	800.75	−6.3	57.14	7.49	−6.86
	(4,542.28–5,938.71)	(705.15–915.13)	(−6.62 to −5.98)*	(49.08–65.09)	(6.54–8.58)	(−7.52 to −6.2)*
Oceania	8,022.49	5,401.42	−1.36	89.66	57.74	−1.51
	(6,733.72–9,456.47)	(4,177.52–6,934.63)	(−1.46 to −1.25)*	(75.17–105.11)	(43.82–75.69)	(−1.63 to −1.39)*
Sub-Saharan Africa						
Western Sub-Saharan Africa	23,722.39	10,333.99	−2.85	270.37	112.97	−2.98
	(20,457.42–26,522.82)	(8,464.8–12,698.94)	(−3.01 to −2.68)*	(233.59–302.71)	(91.45–139.98)	(−3.13 to −2.83)*
Central Sub-Saharan Africa	17,190.39	5,824.18	−3.68	205.31	67.32	−3.79
	(14,295.83–19,921.32)	(4,692.23–7,256.92)	(−3.91 to −3.44)*	(172.34–237.21)	(53.86–84.82)	(−4.03 to −3.55)*
Southern Sub-Saharan Africa	9,199.87	5,401.70	−1.83	103.4	58.42	−1.96
	(8,085.22–10,397.04)	(4,354.85–6,724.74)	(−2.04 to −1.62)*	(90.88–116.64)	(46.73–73.83)	(−2.17 to −1.75)*
Eastern Sub-Saharan Africa	20,278.95	6,616.55	−3.8	251.07	77.78	−3.96
	(17,899.31–22,885.12)	(5,432.15–8,177.81)	(−3.95 to −3.65)*	(222.44–283.85)	(64.3–96.15)	(−4.13 to −3.79)*
South Asia	15,883.22	6,323.59	−3.14	172.69	59.01	−3.64
	(14,426.99–17,540.6)	(5,442.46–7,390.16)	(−3.18 to −3.1)*	(155.2–192.43)	(50.01–70.4)	(−3.69 to −3.59)*
Latin America and Caribbean						
Tropical Latin America	7,941.39	2,209.42	−4.31	88.03	22.03	−4.66
	(6,976.97–9,185.75)	(1,848.74–2,617.33)	(−4.36 to −4.27)*	(77.46–101.91)	(18.15–26.6)	(−4.72 to −4.61)*
Caribbean	7,701.98	4,292.55	−1.93	83.54	44.03	−2.12
	(6,840.74–8,757.76)	(3,460.44–5,257.49)	(−2.24 to −1.63)*	(73.75–94.94)	(35–55.19)	(−2.45 to −1.78)*
Andean Latin America	7,494.76	1,897.99	−4.67	86.84	21	−4.85
	(6,826.26–8,234.08)	(1,496.09–2,363.3)	(−4.75 to −4.59)*	(79.49–94.52)	(16.32–26.6)	(−4.97 to −4.73)*
Central Latin America	5,483.47	1,619.21	−4.1	70.3	18.74	−4.44
	(4,921.46–5,990.18)	(1,291.8–1,968.1)	(−4.15 to −4.05)*	(62.81–76.15)	(15.05–23.06)	(−4.66 to −4.23)*
North Africa and Middle East	9,300.92	2,698.90	−4.17	100.97	26.37	−4.52
	(8,172.62–10,674.7)	(2,312.97–3,160.53)	(−4.31 to −4.03)*	(88.67–115.86)	(22.38–31.51)	(−4.65 to −4.4)*
Central Europe, eastern Europe, and central Asia						
Central Europe	2,310.34	686.73	−4	21.81	4.61	−5.07
	(2,128.87–2,512.92)	(572.5–816.84)	(−4.22 to −3.77)*	(20.28–23.47)	(3.65–5.72)	(−5.61 to −4.52)*
Central Asia	7,015.62	2,603.10	−3.38	71.99	23.22	−3.84
	(6,342.13–7,798.5)	(2,183.17–3,125.26)	(−3.53 to −3.23)*	(64.08–80.08)	(19.04–28.61)	(−4 to −3.69)*
Eastern Europe	1,828.15	648.38	−3.51	17.4	4.66	−4.44
	(1,689.93–2,012)	(548.65–757.92)	(−4.26 to −2.75)*	(16.15–19.32)	(3.8–5.66)	(−5.35 to −3.51)*
High–income regions						
High–income North America	1,046.44	747.61	−1.15	9.7	6.37	−1.41
	(980.5–1,118.82)	(679.94–825.36)	(−1.31 to −0.98)*	(9.28–10.22)	(5.85–6.92)	(−1.65 to −1.18)*
High–income Asia Pacific	819.91	361.05	−2.76	5.41	1.72	−3.88
	(695.02–966.66)	(300.31–440.78)	(−2.97 to −2.56)*	(4.88–6.14)	(1.54–1.91)	(−4.19 to −3.56)*
Australasia	858.92	467.16	−2.04	7.09	3.22	−2.68
	(784.07–943.73)	(401.2–538.67)	(−2.24 to −1.85)*	(6.64–7.66)	(2.69–3.84)	(−2.98 to −2.38)*
Western Europe	819.29	451.62	−2.05	7.21	3.27	−2.72
	(754.79–890.73)	(389.06–515.31)	(−2.12 to −1.97)*	(6.89–7.66)	(2.82–3.79)	(−2.88 to −2.56)*
Southern Latin America	2,643.86	1,028.31	−3.22	27.48	10.56	−3.35
	(2,492.15–2,824.75)	(846.56–1,231.59)	(−3.64 to −2.8)*	(26.16–28.73)	(8.63–12.7)	(−3.67 to −3.03)*

*p < 0.05.

Compared to females, males have higher age-standardized rates of DALY (4,552.18/100,000, 95% UI: 3,865.31/100,000–5,418.67/100,000) and death (46.96/100,000, 95% UI: 39.38/100,000–56.97/100,000). The <28 days age group had the highest age-standardized DALY and death rates of CMM (1,554,055.35/100,000, 95% UI: 1,322,830.84/100,000–1,845,976.9/100,000 and 17,464.36/100,000, 95% UI: 14,860.36/100,000–20,754.84/100,000; Table 1).

Maternal and neonatal disorders were the leading causes of DALY (52.47%) and death (55.95%) attributable to CMM (Supplementary Figure 1). For death, the second and third rankings were respiratory infections and tuberculosis (19.26%) and enteric infections (15.40%), respectively. For DALY, the second and third rankings were respiratory infections and tuberculosis (16.88%) and nutritional deficiencies (15.26%), respectively (Supplementary Figure 1).

### Regional variations

In 2019, Western Sub-Saharan Africa had the highest age-standardized rates of DALY (10,333.99/100,000, 95% UI: 8,464.8/100,000– 12,698.94/100,000) and death (112.97/100,000, 91.45/100,000–139.98/100,000) of CMM followed by Eastern Sub-Saharan Africa (6,616.55/100,000 for DALY and 77.78/100,000 for death) and South Asia (6,323.59/100,000 for DALY and 59.01/100,000 for death; Table 1; Figures 1A,B).

**Figure 2:**
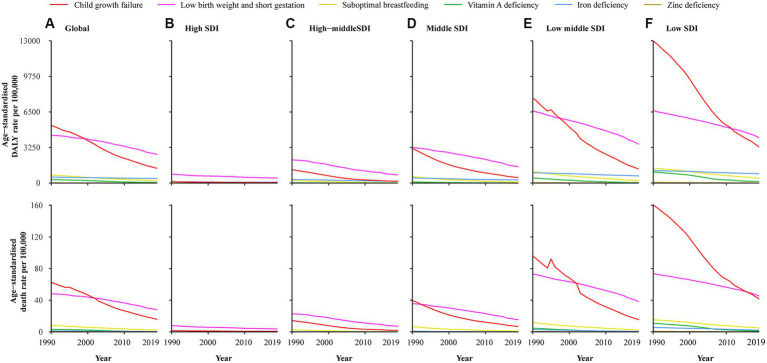
Age-standardized DALY and death rate attributable to CMM among SDI quintiles from 1990 to 2019. **(A)** Global; **(B)** High SDI; **(C)** High-middle SDI; **(D)** Middle-SDI; **(E)** Low middle SDI; **(F)** Low SDI.

The SDI level and burden of CMM demonstrate a clear negative correlation, with the highest age-standardized rates of DALY (8,411.63/100,000, 95% UI: 7,020.74/100,000–10,230.56/100,000) and death (90.03/100,000, 95% UI: 74.34/100,000–110.95/100,000) in the lowest SDI regions. In high SDI regions, both age-standardized rates of DALY (580.86/100,000, 95% UI: 517.96/100,000–649.96/100,000) and death (4.43/100,000, 95% UI: 4/100,000–4.89/100,000) are the lowest (Table 1; Figure 2).

### Global trends

**Figure 3:**
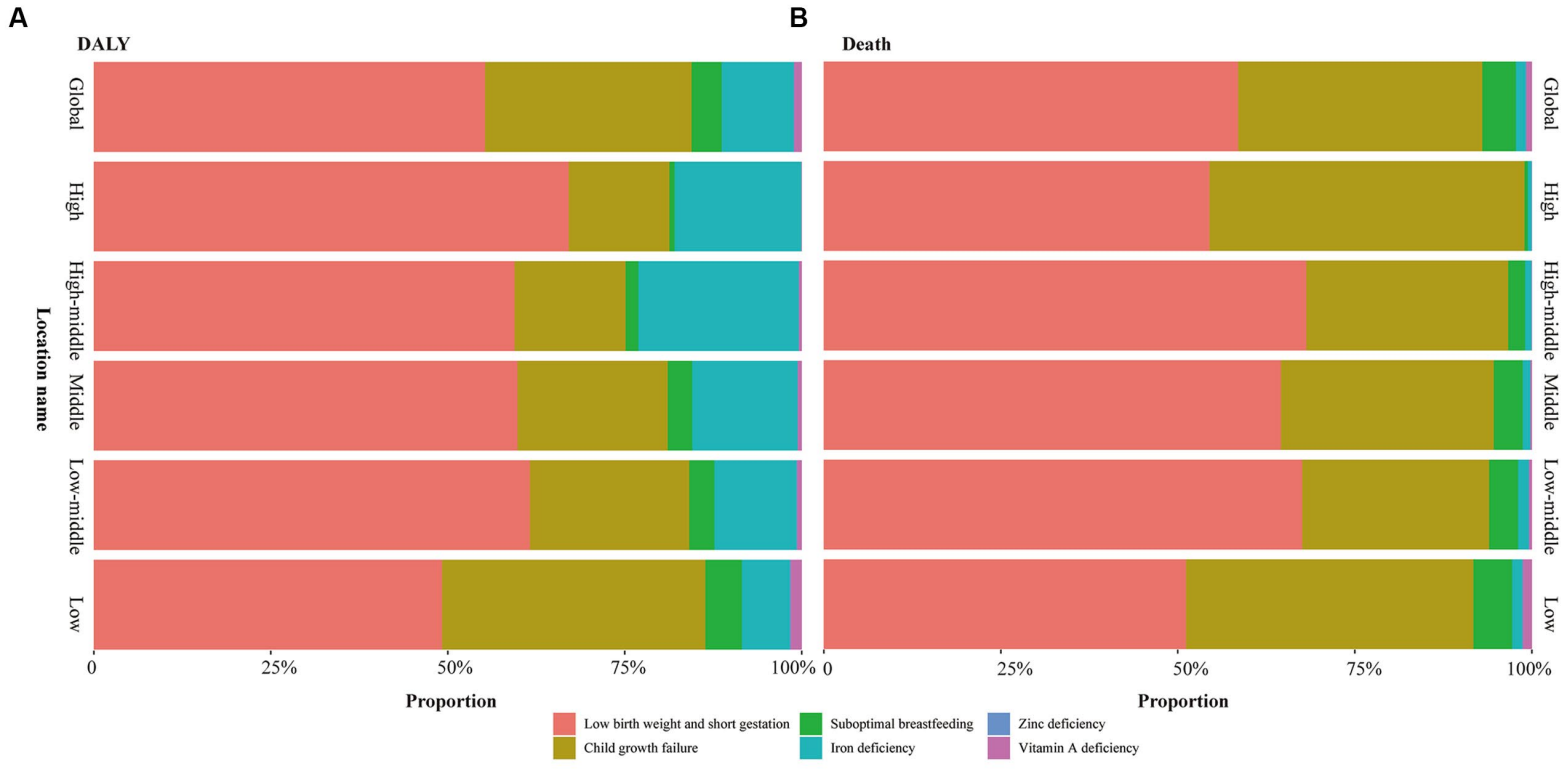
Contribution of each specific risk factor for DALY and death of global and each SDI regions. **(A)** DALY; **(B)** Death.

Globally, the age-standardized DALY and death rates showed significantly declining trends from 1990 to 2019 (AAPC = −2.92, 95% CI:2.97% to −2.87% and AAPC = −3.19, 95% CI: −3.27 to 3.12%). Furthermore, significantly declining trends were also observed in all of six specific risk factors (Table 1; Supplementary Figure 2). The age-standardized DALY and death rates attributed to CMM decreased from 1990 to 2019 in almost all countries and regions, age groups, and SDI quintiles (Figures 1C,D, 3).

### Risk factors

In 2019, low birth weight and short gestation and was primary type of the CMM globally, which accounted for 55.25% and 58.51% of the total DALYs and deaths cases (Figure 3), respectively. In 2019, the global age-standardized DALY and death rates of low birth weight and short gestation were 2,604.41/100,000 (2,232.22/100,000–308,5.68) and 27.87/100,000 (23.73/100,000–33.09/100,000), respectively (Table 1). Subsequently, the second and third rankings were child growth failure and iron deficiency, respectively (Figure 3).

**Figure 4:**
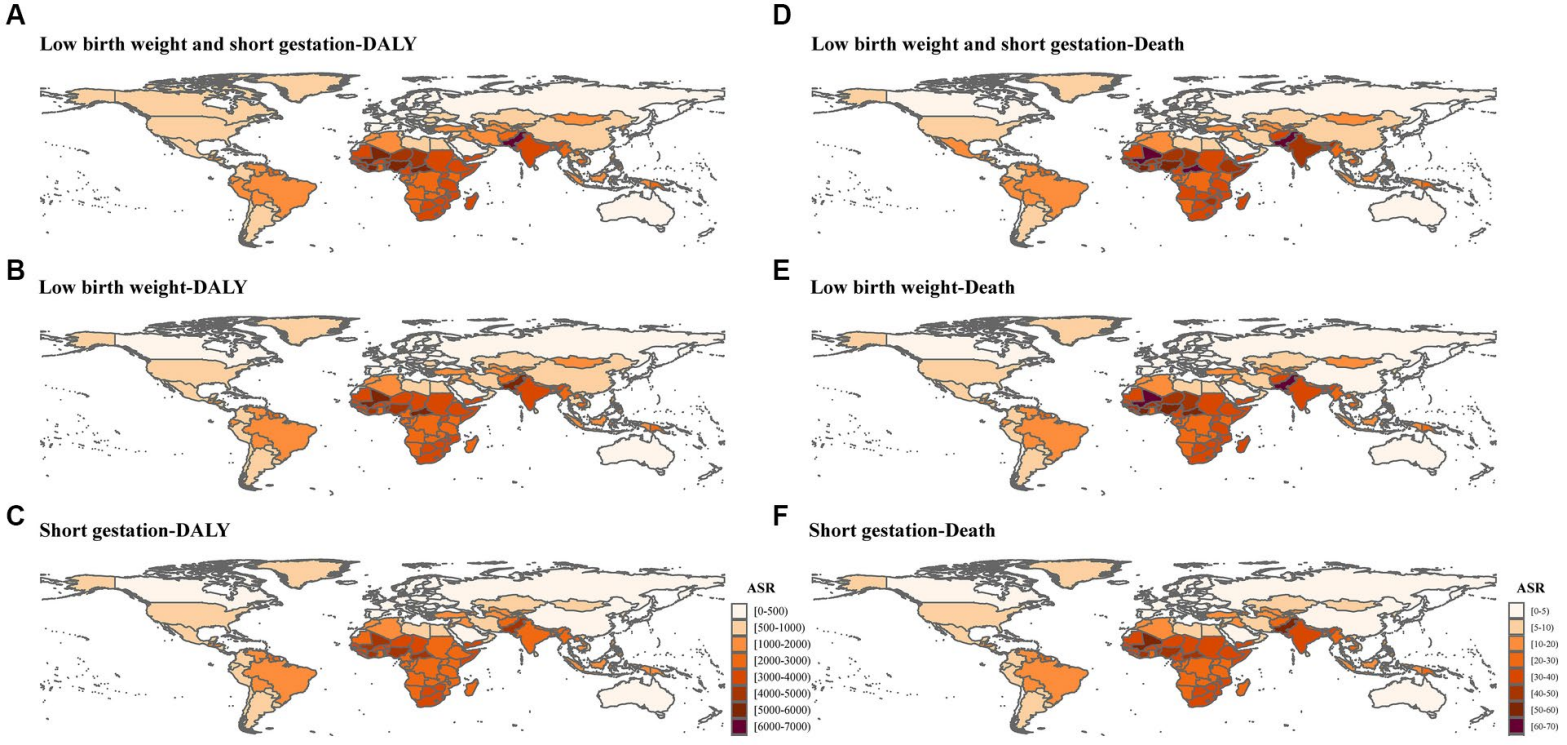
Global burden attributable to low birth weight and short gestation in 2019. **(A)** Age-standardized DALY rate of low birth weight and short gestation; **(B)** Age-standardized DALY rate of low birth weight; **(C)** Age-standardized DALY rate of short gestation; **(D)** ASDR of low birth weight and short gestation; **(E)** ASDR of low birth weight; **(F)** ASDR of short gestation.

In the past three decades, though, low birth weight and short gestation has been on a downward trend in most countries and regions (Supplementary Figure 3). It still caused heavy burden in Sub-Saharan Africa and low SDI regions, and there is an upward trend in Dominica, Guam and Zimbabwe (Figure 4; Supplementary Figure 3).

### Sociodemographic factors

**Figure 5:**
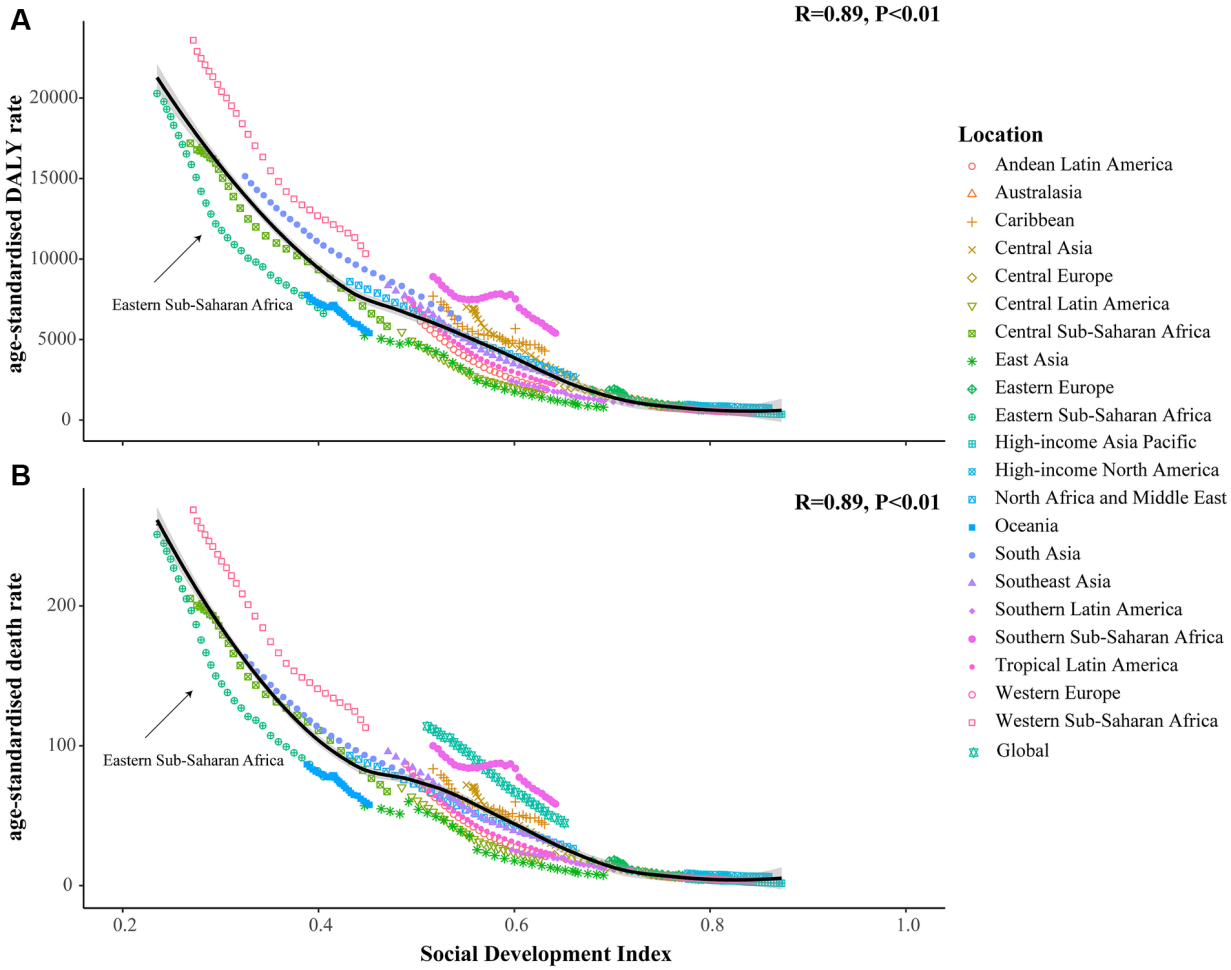
The correlation between SDI scores and the age-standardized rates of DALY and death in 21 GBD regions. **(A)** The correlation between SDI scores and the age-standardized DALY rate; **(B)** The correlation between SDI scores and ASDR. The horizontal axis represents the score of the social development index, ranging from 0 for low to 1 for high. Meanwhile, the vertical axis represents the age-standardized rates of DALY and death, with distinct shapes denoting different GBD regions.

We found that the age-standardized DALY and death rates were negatively correlated with the SDI scores in the 21 GBD regions, with correlation coefficients of 0.89 (*p* < 0.05; Figure 5). The GLM results indicated that the GDP *per capita* (*β* = −0.1968 for age-standardized DALY rate, *β* = −0.271 for ASDR), current health expenditure *per capita* (*β* = −0.2841 for age-standardized DALY rate, *β* = −0.2889 for ASDR), physicians per 1,000 people (*β* = −0.242 for age-standardized DALY rate, *β* = −0.2943 for ASDR) were contributed to reduce the burden attributable to CMM (Table 2).

**Table 2:** The correlation between sociodemographic factors and DALY and death rates attributable to CMM.

	Age-standardized DALY rate			Age-standardized death rate		
	*β*	*Sd*	*P*	*β*	*Sd*	*P*
Log *per capita* gross domestic product	−0.1968	0.0225	<0.01**	−0.271	0.0275	<0.01**
Log *per capita* current health expenditure	−0.2841	0.0182	<0.01**	−0.2889	0.0222	<0.01**
Urban population (% of total population)	0.0008	0.0007	*0.254*	0.0016	0.0009	0.0682
Log Population	−0.0042	0.0048	*0.381*	−0.0005	0.0058	0.9299
Log Population density	−0.0591	0.0068	<0.01**	−0.0754	0.0084	<0.01**
Log Physicians (per 1,000 people)	−0.242	0.0102	<0.01**	−0.2943	0.0124	<0.01**
(Intercept)	11.2035	0.1388	<0.01**	7.1373	0.17	<0.01**

**p < 0.01.

## Discussion

In this study, we examined the global burden and trends of disease attributable to CMM during the past 30 years. We also explored the sociodemographic factors associated with burden attributable to CMM. Our findings showed that global age-standardized DALY and death rates have continuously decreased. However, there are still substantial DALYs and death cases occurring annually, especially in infants aged <28 days, Sub-Saharan Africa, and low SDI regions. The burden attributable to CMM was significantly negatively correlated with SDI scores. Higher GDP *per capita*, current health expenditure *per capita*, and physicians were found to significantly contribute to reducing the burden attributable to CMM.

The downward trend observed in the burden attributed to CMM is a result of joint efforts made by people worldwide. Previous study has summarized evidence-based interventions that have been effective in reducing CMM over the past decade, including providing preventive zinc, preventive small-quantity lipid-based nutrient supplements, maternal and child multiple micronutrient powder (MMN), maternal calcium vitamin A supplementation, promotion of breastfeeding, farm input subsidies, appropriate complementary feeding and management of moderate and severe acute malnutrition, etc. (7, 29, 30). These measures have considerably improved the nutritional health of children and pregnant women, thereby decreasing the disease burden caused by CMM (31–33). For instance, during food crises, community-based supplementary food is provided and locally produced complementary and therapeutic foods are used to handle acutely malnourished children (34, 35). Additionally, some newly launched interventions, such as preventive low-dose lipid supplements for children aged 6–23 months, have demonstrated with positively impacts on children’s growth and development (36–38). Indirect nutrition strategies implemented by the healthcare sector or other sectors, such as malaria prevention, pre-pregnancy care, water, sanitation, and hygiene improvement, have also contributed significantly to reducing the disease burden of CMM (39–42). Between 1980 and 2000, the mortality rate of babies in the first month of life from 2 months to 5 years decreased by 1/3, while the neonatal mortality rate decreased by about 1/4 (43).

Compared with previous studies in specific country (11, 12), this study offers a more comprehensive perspective for exploring global trends. Furthermore, it is capable of examining the most vulnerable populations and regions. We found that newborns, Sub-Saharan Africa, and regions with a low level of SDI require more attention. For infants <28 days, their bodies are adapting to the external environment. During this process, infants with poor physical conditions may become sick or die because they cannot adjust to the surroundings. Research has shown that although the neonatal period only lasts for 28 days, it accounts for 38% of all deaths among children under the age of 5 (43). Especially in Sub-Saharan Africa, which takes the highest neonatal mortality rates, and the neonatal mortality rate among children under the age of five is 26%. Among 18 countries, of 14 have a neonatal mortality rate of 45 per 1,000 people (43). With a few exceptions, countries in Sub-Saharan Africa have accomplish little progress in reducing neonatal deaths over the past decade (43). For a long time, agriculture and animal husbandry production in Sub-Saharan Africa has lagged behind, leading to low output of agricultural products, and a growth rate of food production that is much lower than that of population growth. Consequently, inadequate food supply has become a common problem in Sub-Saharan Africa. For the past two decades, these regions have consistently had the highest levels of hunger with high GHI score. In all of Sub-Saharan Africa, there exists the highest level of undernourishment (44) with undernutrition hotspots (45). The same situation has also been observed in India, malnutrition served as the predominant risk factor for death in children under the age of 5 in whole region (11).

In previous GBD studies, SDI as a comprehensive index measuring the developmental status of a country or region, is frequently used to analyze its correlation with the burden of disease (18, 19, 46). It measures social development and provides an overall assessment of a country or region’s level of development, including *per capita* GDP, the average length of education of adults, and the fertility rate of women under the age of 25 (47). As countries and regions increase sustainable development indicators through higher levels of education, particularly for women, we can look forward to progress on these risks (48). While public health interventions such as immunization, breastfeeding, and improved sanitation can reduce neonatal and late neonatal deaths, moreover, the provision of personalized clinical care also supply as a significant decrease in early neonatal (and maternal) deaths (43).

Our results reveal that national economic level, medical expenditure, and the allocation of medical resources are crucial factors influencing the disease burden attributed to CMM. Countries with higher *per capita* GDP have better economic conditions, greater availability of health resources, improved accessibility and diversity of food, and a reduced likelihood of malnutrition among their residents compared to countries with low *per capita* GDP (48). Countries with higher *per capita* GDP also have greater capacity to achieve universal healthcare, reduced inequality in health status and determinants among residents, and more access to medical services (49, 50). Research indicates that every 1% increase in *per capita* government health expenditure increases life expectancy by 0.0169%, and every 1% increase in *per capita* private health expenditure increases life expectancy by 0.0032% (51). GDP and domestic health expenditure are common macroeconomic indicators used to measure living standards and public health policies. The number of doctors per thousand population is the main index used to measure the development of medical human resources in the region. A larger number of doctors per thousand population means more adequate medical human resources, greater possibilities for residents to get medical help, and a reduced incidence of disease burden (52). Therefore, expanding coverage of care during childbirth and early postpartum can significantly reduce neonatal mortality in this environment, benefiting the poorest and underserved groups. In areas with heavy burdens, increasing investment in medical resources and allocation of medical resources *per capita* can effectively reduce the burden attributed to CMM.

### Limitations

This study retrieved data from public database of GBD, and the data collected from various sources, potentially containing errors, anomalies, or uncertainties, which may introduce some bias. Nevertheless, the GBD database still remains one of the most comprehensive and widely used database. Furthermore, strengthening collaboration and data sharing, especially among governments, across regions, and among scholars, is also a crucial pathway to enhance data quality and accessibility. Secondly, considering the latest update in GBD as of 2019, our study was unable to account for the long term impacts of the pandemic and regional conflicts on CMM, as well as their subsequent effects (53, 54). In the future, by obtaining the latest update by GBD, along with more comprehensive and relevant data, and conducting in-depth research, we can explore these issues, thereby obtaining more valuable and worthwhile findings.

## Conclusion

Despite the continuous decrease in global age-standardized DALY and death rates attributable to CMM, there are still significant DALYs and deaths occurring annually, especially among infants aged <28 days, in Sub-Saharan Africa, and in low SDI regions. Further research could examine the specific factors contributing to CMM in high burden regions, aiming to develop region-specific interventions. Among the CMM, low birth weight and short gestation has become the primary risk factors globally. The burden attributable to CMM showed a significant negative correlation with SDI scores, while higher *per capita* GDP, *per capita* current health expenditure, and physicians per 1,000 people significantly contributed to reducing the burden, it provides the insights into effective resource allocation and healthcare policies. Therefore, our study offers valuable insights to guide targeted interventions and shape future research endeavors in addressing the burden of CMM and enhancing maternal and child health globally.

## Data availability statement

All data are open-access and are available from The Global Burden of Disease 2019 (https://vizhub.healthdata.org/gbd-results/).

## Author contributions

RL: Conceptualization, Data curation, Formal analysis, Methodology, Resources, Validation, Writing – original draft, Writing – review & editing. LP: Data curation, Formal analysis, Writing – original draft. FL: Writing – original draft. QS: Supervision, Writing – review & editing.

## Footnotes

^1^https://vizhub.healthdata.org/gbd-results/
