# Supplementary material for: Global disability-adjusted life years and deaths attributable to child and maternal malnutrition from 1990 to 2019
Source: Front Public Health. 2024 Jan 18;12:1323263. doi: 10.3389/fpubh.2024.1323263 (PMC10830744; doi:10.3389/fpubh.2024.1323263)
Supplement: Supplementary file 1 [file Data_Sheet_1.doc]

**Supplementary Table 1. The number of DALY and death attributable to CMM in 1990 and 2019**

|  | **DALY (95% UI)** | | **Death (95% UI)** | |
| --- | --- | --- | --- | --- |
|  |  |  |  |  |
|  | **1990** | **2019** | **1990** | **2019** |
|  |  |  |  |  |
|  | Number | Number | Number | Number |
| **Global** | 665,322,525 | 294,779,309 | 7,263,624 | 2,937,804 |
|  | (612,705,770-725,198,458) | (252,994,742-349534,865) | (6,677,775-7,942,657) | (2,489,636-3,512,073) |
| **Gender** |  |  |  |  |
| Male | 350,090,385 | 155,219,568 | 3,835,364 | 1,578,852 |
|  | (318,866,878-382,212,949) | (131,999,096-184,877,020) | (3,476,268-4,197,691) | (1,323,010-1,916,747) |
| Female | 315,232,140 | 139,559,741 | 3,428,260 | 1,358,952 |
|  | (288,816,370-343,654,007) | (120,607,644-164,272,415) | (3,129,866-3,757,745) | (1,159,658-1,611,748) |
| **Age group** |  |  |  |  |
| <28 days | 275,847,988 | 158,798,848 | 3,101,670 | 1,784,570 |
|  | (256,875,265-296,086,082) | (135,171,513-188,628,419) | (2,888,189-3,329,503) | (1,518,484-2,120,803) |
| 28-364 days | 192,728,757 | 53,363,896 | 2,151,119 | 578,035 |
|  | (172,172,050-215,096,496) | (43,470,801-66,766,477) | (1,922,841-2,405,869) | (465,482-728,826) |
| 1-4 years | 160,940,231 | 42,113,492 | 1,784,384 | 414,346 |
|  | (139,384,194-186,355,023) | (33,827,693-52,022,272) | (1,540,858-2,085,541) | (323,810-528,191) |
| 5-14 years | 12,293,856 | 12,039,907 | 30,363 | 7,127 |
|  | (9,271,672-16,113,107) | (8,756,513-16,393,097) | (21,577-44,207) | (5,665-8,703) |
| 15-49 years | 17,202,180 | 19,506,531 | 99,360 | 55,339 |
|  | (12,812,733-22,066,981) | (14,692,894-25,054,326) | (51,223-145,396) | (28,349-83,917) |
| 50-69 years | 4,535,693 | 6,339,940 | 33,023 | 21,121 |
|  | (3,397,781-5,921,136) | (4,722,903-8,347,779) | (25,634-44,369) | (18,748-24,015) |
| 70+ years | 1,773,820 | 2,616,696 | 63,705 | 77,266 |
|  | (1,458,420-2,142,770) | (2,104,030-3,291,235) | (53,773-73,576) | (67,714-84,057) |
| **SDI region** |  |  |  |  |
| High SDI | 6,866,802 | 4,072,426 | 58,707 | 33,432 |
|  | (6,256,687-7,600,292) | (3,530,733-4,701,970) | (55,202-62,834) | (29,646-36,317) |
| High-middle SDI | 38,234,151 | 10,467,150 | 383,407 | 77,695 |
|  | (34,378,867-42,340,741) | (8,893,179-12,138,278) | (343,322-425,640) | (68,297-89,015) |
| Middle SDI | 142,352,019 | 42,728,800 | 1,528,641 | 394,617 |
|  | (130,149,112-155,113,695) | (36,773,401-50,084,670) | (1,401,634-1,664,640) | (340,794-464,180) |
| Low-middle SDI | 265,687,627 | 95,019,123 | 2,919,489 | 926,268 |
|  | (244,662,526-290,439,475) | (81,860,260-111,543,400) | (2,669,603-3,205,771) | (790,895-1,099,056) |
| Low SDI | 211,866,375 | 142,315,723 | 2,369,926 | 1,503,972 |
|  | (190,125,704-235,386,079) | (118,402,707-173,810,301) | (2,116,607-2,640,620) | (1,229,323-1,857,479) |
| **Type of Cause** |  |  |  |  |
| Suboptimal breastfeeding | 47,368,655 | 13,145,101 | 532,453 | 146,322 |
|  | (34,962,431-59,335,024) | (9,346,954-17,614,516) | (393,563-668,370) | (103,706-197,044) |
| Iron deficiency | 29,741,477 | 31,263,125 | 73,461 | 42,349 |
|  | (20,506,000-41,287,216) | (21,271,982-43,987,242) | (27,235-117,529) | (15,000-70,283) |
| Vitamin A deficiency | 20,127,856 | 3,297,057 | 207,555 | 23,850 |
|  | (3,572,772-40,332,831) | (1,347,266-5,593,876) | (19,446-443,061) | (2,667-49,862) |
| Zinc deficiency | 1,574,181 | 258,813 | 17,924 | 2,806 |
|  | (528,156-3,091,082) | (66,897-597,019) | (5,907-35,228) | (697-6,527) |
| Child growth failure | 336,210,400 | 89,759,926 | 3,903,004 | 1,064,706 |
|  | (297,805,591-380,611,304) | (72,602,623-111,739,391) | (3,449,044-4,422,977) | (867,352-1,311,437) |
| Low birth weight and short gestation | 286,456,377 | 170,087,071 | 3,167,605 | 1,805,482 |
|  | (266,580,199-307,079,118) | (145,912,388-201,254,339) | (2,947,282-3,400,387) | (1,537,291-2,143,500) |
| **Southeast Asia, east Asia, and Oceania** |  |  |  |  |
| Southeast Asia | 52,709,828 | 14,480,938 | 578,695 | 149,593 |
|  | (47,780,071-58,452,906) | (12,341,041-16,855,328) | (522,999-643,509) | (127,182-173,706) |
| East Asia | 63,006,870 | 7,641,560 | 670,252 | 62,050 |
|  | (54,603,849-71,458,726) | (6,614,937-8,775,482) | (573,524-765,541) | (54,647-70,388) |
| Oceania | 790,253 | 998,838 | 8,542 | 10,475 |
|  | (660,656-933,878) | (769,476-1,291,909) | (7,087-10,174) | (7,822-13,821) |
| **Sub-Saharan Africa** |  |  |  |  |
| Western Sub-Saharan Africa | 89,495,314 | 76,387,842 | 1,005,213 | 823,900 |
|  | (77,411,764-99,714,148) | (62,391,560-94,074,249) | (869,689-1,123,915) | (665,430-1,028,277) |
| Central Sub-Saharan Africa | 19,159,387 | 11,536,438 | 215,745 | 123,391 |
|  | (15,958,540-22,293,339) | (9,292,598-14,576,625) | (180,088-251,111) | (98,185-157,993) |
| Southern Sub-Saharan Africa | 6,540,050 | 4,300,296 | 72,147 | 45,549 |
|  | (5,743,046-7,405,276) | (3,467,400-5,359,148) | (63,129-81,791) | (36,231-57,974) |
| Eastern Sub-Saharan Africa | 74,695,516 | 41,288,570 | 848,540 | 445,244 |
|  | (65,578,305-84,309,193) | (33,597,387-51,572,360) | (745,120-956,327) | (358,262-561,147) |
| South Asia | 257,081,572 | 103,633,972 | 2,778,990 | 949,135 |
|  | (233,439,294-283,380,311) | (88,902,150-121,101,924) | (2,504,979-3,074,353) | (804,539-1,131,228) |
| **Latin America and Caribbean** |  |  |  |  |
| Tropical Latin America | 13,419,134 | 3,779,690 | 144,611 | 36,536 |
|  | (11,776,015-15,540,017) | (3,188,578-4,448,797) | (126,582-168,285) | (30,471-43,644) |
| Caribbean | 3,248,479 | 1,723,595 | 35,126 | 17,544 |
|  | (2,884,549-3,685,361) | (1,395,809-2,102,156) | (30,972-39,921) | (13,959-21,943) |
| Andean Latin America | 4,081,498 | 1,196,512 | 44,991 | 12,871 |
|  | (3,716,559-4,494,843) | (942,928-1,490,849) | (40,918-49,358) | (9,956-16,352) |
| Central Latin America | 12,303,509 | 3,546,005 | 140,539 | 40,392 |
|  | (11,017,049-13,482,851) | (2,845,159-4,291,859) | (125,474-153,707) | (32,455-49,596) |
| North Africa and Middle East | 50,287,427 | 15,931,115 | 548,818 | 152,881 |
|  | (44,190,830-57,689,133) | (13,664,371-18,677,904) | (480,985-630,946) | (129,408-183,006) |
| **Central Europe, eastern Europe, and central Asia** |  |  |  |  |
| Central Europe | 2,024,053 | 497,827 | 17,901(16,618-19,270) | 2,658(2,146-3,263) |
|  | (1,844,765-2,225,339) | (412,573-596,986) |  |  |
| Central Asia | 6,456,267 | 2,403,845 | 67,713 | 21,268 |
|  | (5,807,379-7,183,628) | (2,016,429-2,886,523) | (60,243-75,336) | (17,411-26,209) |
| Eastern Europe | 2,880,051 | 962,792 | 25,263 | 5,340 |
|  | (2,622,626-3,185,691) | (801,616-1,151,669) | (23,468-28,024) | (4,391-6,413) |
| **High-income regions** |  |  |  |  |
| High-income North America | 2,441,420 | 1,904,608 | 22,429 | 17,255 |
|  | (2,275,682-2,626,426) | (1,699,575-2,134,420) | (21,416-23,589) | (15,755-18,531) |
| High-income Asia Pacific | 1,068,246 | 512,263 | 5,432 | 2,489 |
|  | (871,307-1,321,791) | (404,689-654,252) | (4,944-6,166) | (2,098-2,740) |
| Australasia | 141,645 | 102,219 | 1,097 | 673 |
|  | (128,004-157,113) | (87,633-118,670) | (1,030-1,185) | (578-784) |
| Western Europe | 2,164,801 | 1,417,198 | 17,926 | 12,778 |
|  | (1,958,317-2,395,262) | (1,203,376-1,662,151) | (17,139-19,003) | (10,733-14,405) |
| Southern Latin America | 1,327,203 | 533,185 | 13,654 | 5,782 |
|  | (1,250,543-1,417,740) | (443,262-634,364) | (12,990-14,274) | (4,902-6,814) |


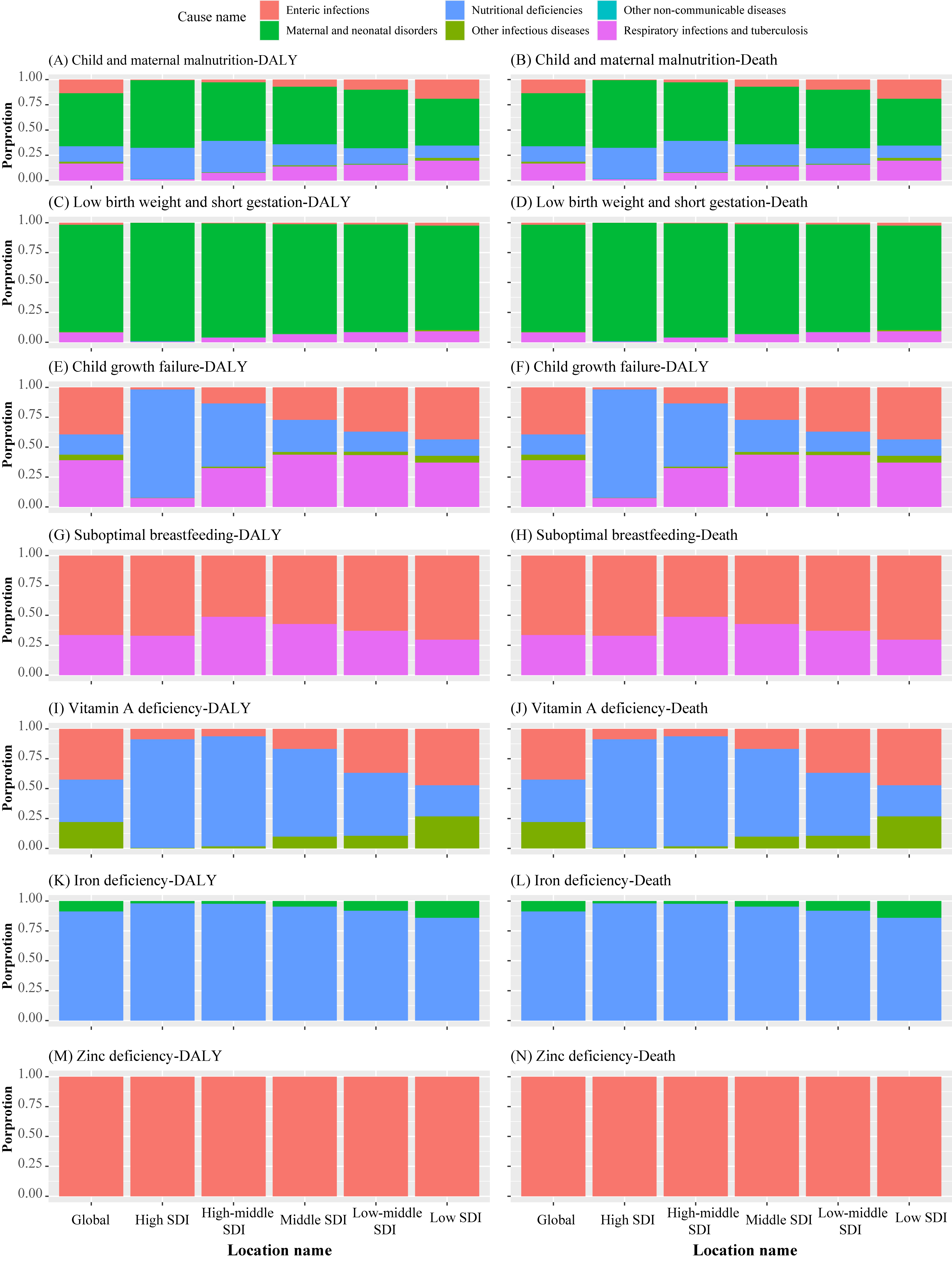


Supplementary Figure 1. The proportion of causes attributable to total CMM and each specific risk factors.


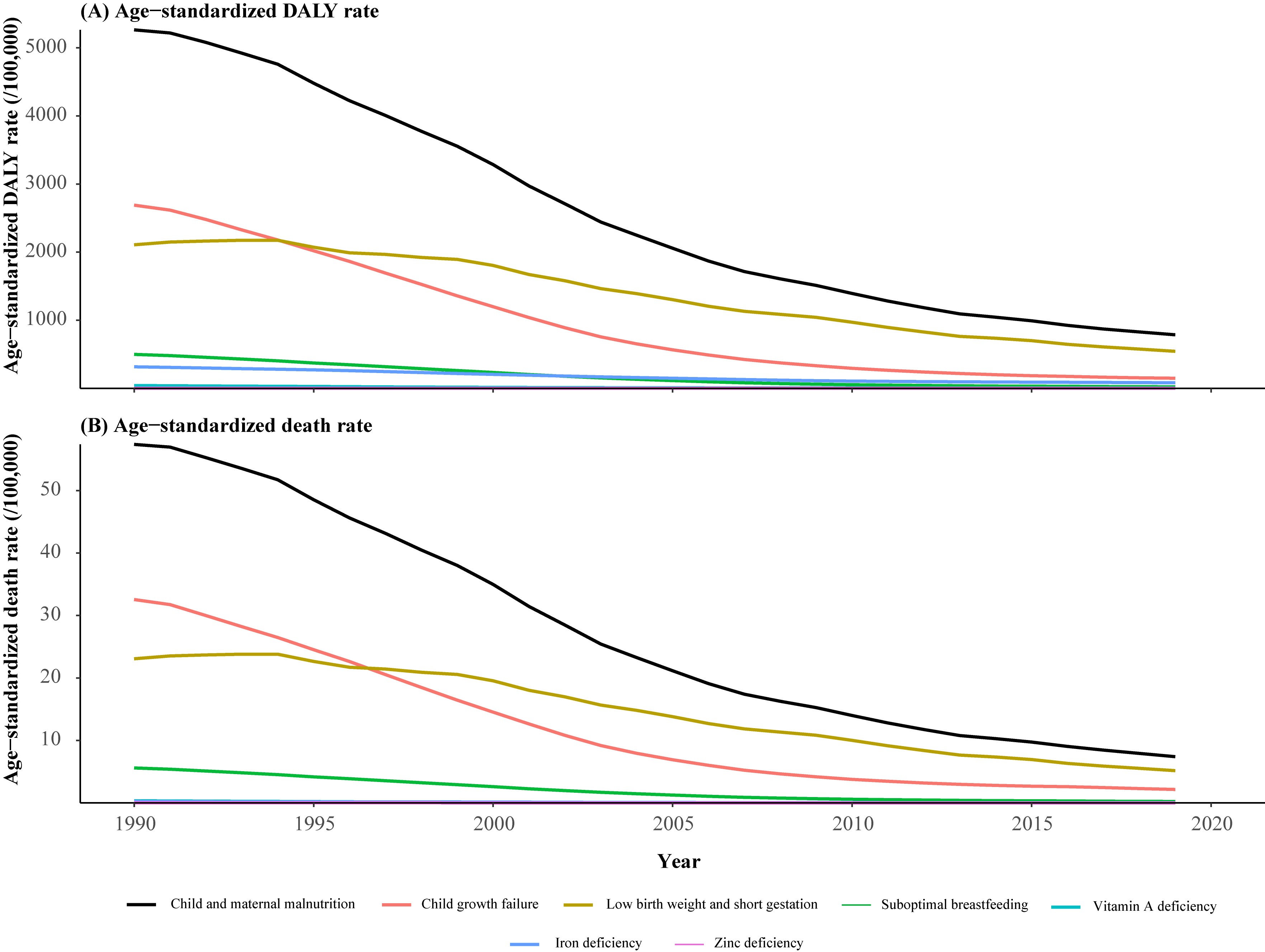


Supplementary Figure 2. Global trends of age-standardized DALY and death rates of six specific risk factors.


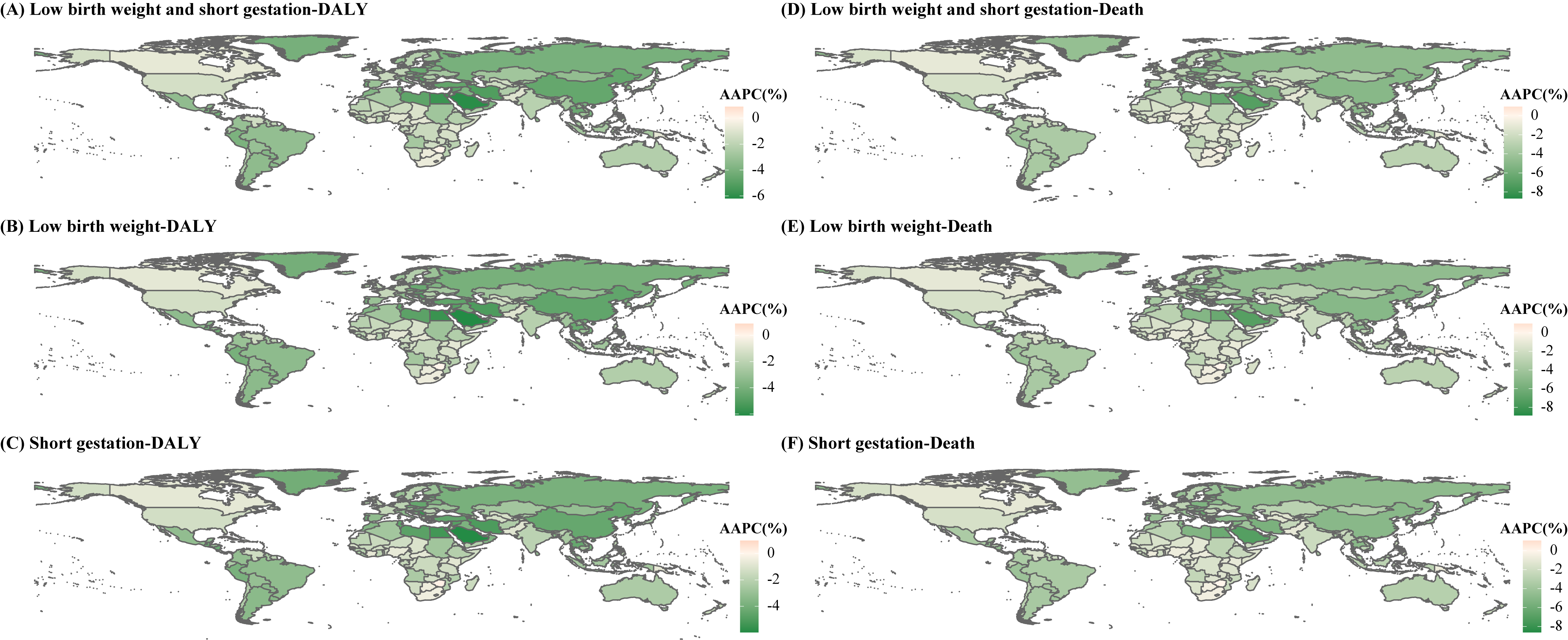
Supplementary Figure 3. The average annual percentage change of age-standardised rates of low birth weight and short gestation from 1990 to 2019.
